# The relationship between endogenous thymidine concentrations and [^18^F]FLT uptake in a range of preclinical tumour models

**DOI:** 10.1186/s13550-016-0218-3

**Published:** 2016-08-11

**Authors:** Kathrin Heinzmann, Davina Jean Honess, David Yestin Lewis, Donna-Michelle Smith, Christopher Cawthorne, Heather Keen, Sandra Heskamp, Sonja Schelhaas, Timothy Howard Witney, Dmitry Soloviev, Kaye Janine Williams, Andreas Hans Jacobs, Eric Ofori Aboagye, John Richard Griffiths, Kevin Michael Brindle

**Affiliations:** 1 Cancer Research UK Cambridge Institute, University of Cambridge, Cambridge, UK; 2 Wolfson Molecular Imaging Centre, Manchester Pharmacy School, University of Manchester, Manchester, UK; 3 Personalised Healthcare and Biomarkers, AstraZeneca, Alderley Park, Macclesfield, UK; 4 Department of Radiology and Nuclear Medicine, Radboud University Medical Centre, Nijmegen, The Netherlands; 5 European Institute for Molecular Imaging (EIMI), Westfälische Wilhelms-Universität (WWU), University Hospital of Münster, Münster, Germany; 6 Comprehensive Cancer Imaging Centre, Imperial College London, London, UK; 7 CRUK-EPSRC Cancer Imaging Centre in Cambridge and Manchester, Cambridge, UK; 8 Present address: Comprehensive Cancer Imaging Centre, Imperial College London, London, UK; 9 Present address: Positron Emission Tomography Research Centre, University of Hull, Hull, UK; 10 Present address: UCL Centre for Advanced Biomedical Imaging, University College London, London, UK; 11 Cancer Research UK Cambridge Institute, Li Ka Shing Centre, Robinson Way, Cambridge, CB2 0RE UK

**Keywords:** [^18^F]Fluorothymidine, Plasma, Tumour, Thymidine, Preclinical PET

## Abstract

**Background:**

Recent studies have shown that 3′-deoxy-3′-[^18^F] fluorothymidine ([^18^F]FLT)) uptake depends on endogenous tumour thymidine concentration. The purpose of this study was to investigate tumour thymidine concentrations and whether they correlated with [^18^F]FLT uptake across a broad spectrum of murine cancer models. A modified liquid chromatography-mass spectrometry (LC-MS/MS) method was used to determine endogenous thymidine concentrations in plasma and tissues of tumour-bearing and non-tumour bearing mice and rats. Thymidine concentrations were determined in 22 tumour models, including xenografts, syngeneic and spontaneous tumours, from six research centres, and a subset was compared for [^18^F]FLT uptake, described by the maximum and mean tumour-to-liver uptake ratio (TTL) and SUV.

**Results:**

The LC-MS/MS method used to measure thymidine in plasma and tissue was modified to improve sensitivity and reproducibility. Thymidine concentrations determined in the plasma of 7 murine strains and one rat strain were between 0.61 ± 0.12 μM and 2.04 ± 0.64 μM, while the concentrations in 22 tumour models ranged from 0.54 ± 0.17 μM to 20.65 ± 3.65 μM. TTL at 60 min after [^18^F]FLT injection, determined in 14 of the 22 tumour models, ranged from 1.07 ± 0.16 to 5.22 ± 0.83 for the maximum and 0.67 ± 0.17 to 2.10 ± 0.18 for the mean uptake. TTL did not correlate with tumour thymidine concentrations.

**Conclusions:**

Endogenous tumour thymidine concentrations alone are not predictive of [^18^F]FLT uptake in murine cancer models.

**Electronic supplementary material:**

The online version of this article (doi:10.1186/s13550-016-0218-3) contains supplementary material, which is available to authorized users.

## Background

The nucleoside analogue, 3′-deoxy-3′-[^18^F]fluorothymidine ([^18^F]FLT) was proposed by Shields et al. [1] as a positron emission tomography (PET) tracer for imaging cell proliferation. [^18^F]FLT is phosphorylated, and consequently trapped in cells, by thymidine kinase 1 (TK1), whose expression reaches a maximum in the late G1 and S phases of the cell cycle [2]. However, although [^18^F]FLT has been well characterized as a tracer, not all tumours take it up and it does not yet have an established clinical role [3]. One of the aims of the IMI QuIC-ConCePT Consortium^1^ was to assess whether [^18^F]FLT uptake measures can be qualified as early imaging biomarkers for predicting response or resistance to therapy, by direct correlation with accepted histopathological metrics of response in a wide range of preclinical tumour models.

Previous studies have reported variability in tumour [^18^F]FLT uptake [4–7] and have linked it with specific tumour properties that are independent of tumour cell proliferation. These include the expression and activity of the plasma membrane nucleoside transporters as well as the activity of TK1, the first enzyme of the DNA salvage pathway. Zhang et al. [5] measured free thymidine concentrations in six xenograft lines, including three variants of one line, and showed evidence for a correlation between [^18^F]FLT uptake and tumour thymidine concentrations, which was also observed by Schelhaas et al. [6] but not by McKinley et al. [7]. Zhang et al. also administered exogenous thymidine, raising plasma thymidine concentrations, and observed a decrease in tumour [^18^F]FLT uptake, suggesting that thymidine and [^18^F]FLT compete as substrates for TK1. Measurement of plasma thymidine concentrations may, therefore, be a critical parameter in deciding whether a patient would be appropriate for [^18^F]FLT PET scanning.

We describe here a modified thymidine assay, based on the method of Li et al. [8], which we used at a single centre (CRUK Cambridge Institute (CI)) to measure thymidine concentrations in plasma, tumour and tissue samples from multiple European research centres in order to address the question of whether the negative correlation between [^18^F]FLT uptake and tumour thymidine concentrations observed previously in smaller studies was valid across a wider range of tumour models. We also examined the variability of thymidine concentrations in plasma and other normal tissue between different murine strains and whether tumours themselves altered plasma thymidine concentration in their hosts.

## Methods

### Contributors

Plasma and tumour samples were analysed from six centres, five participating centres in the IMI QuIC-ConCePT Consortium and one additional centre. The participating institutions were the CRUK Cambridge Institute, Cambridge, UK (CI); AstraZeneca, Alderley Park, Macclesfield, UK (AZ); Imperial College London, UK (IC); Westfälische Wilhelms-Universität, Münster, Germany (WWU) and Radboud University Medical Centre, Nijmegen, The Netherlands (Radboudumc). The additional centre was the Wolfson Molecular Imaging Centre Manchester, UK (WMIC). All procedures performed were carried out in compliance with the national laws on the use of animals in research in the UK (The Animals (Scientific Procedures) Act 1986), Germany and the Netherlands, and with local ethical approval, and also in compliance with the QuIC-ConCePT Ethical Guidelines, based on the NCRI Guidelines for the welfare and use of animals in cancer research [9]. The tumour models used in the study are described in Table 1.

**Table 1:** Tumour models

	Tumour	Type	Origin	Model type	Host (supplier)
CRUK Cambridge Institute (CI)	AsPC-1	Pancreatic	Human	Xenograft^a^	CB17 SCID mouse (Charles River)
	MiaPaCa-2	Pancreatic	Human	Xenograft^a^	CB17 SCID mouse (Charles River)
	PancTu I	Pancreatic	Human	Xenograft^a^	CB17 SCID mouse (Charles River)
	Colo-357	Pancreatic	Human	Xenograft^a^	CB17 SCID mouse (Charles River)
	K8484^b^	Pancreatic	Murine	Syngeneic allograft^a^	PC mouse (p53^R172H^; Pdx1-Cre) (CI)
	KPC^c^	Pancreatic	Murine	Spontaneous^d^	KPC mouse (Kras^G12D^; p53^R172H^; Pdx1-Cre) (CI)
AstraZeneca (AZ)	PC9	NSCLC	Human	Xenograft^a^	CB17 SCID mouse (AstraZeneca)
	A431	Squamous carcinoma	Human	Xenograft^a^	ONU mouse (AstraZeneca)
	H1975	NSCLC	Human	Xenograft^a^	ONU mouse (AstraZeneca)
Imperial College London (IC)	HCT116	Colorectal	Human	Xenograft^a^	BALB/c nu mouse (Charles River)
WWU Münster	A549	NSCLC	Human	Xenograft^a^	NMRI nu mouse (Janvier)
	HTB56	NSCLC	Human	Xenograft^a^	NMRI nu mouse (Janvier)
	EBC1	NSCLC	Human	Xenograft^a^	NMRI nu mouse (Janvier)
	H1975	NSCLC	Human	Xenograft^a^	NMRI nu mouse (Janvier)
WMIC Manchester	MDA-MB-231-MFP	Breast	Human	Xenograft^e^	CBA nu/nu mouse (University of Manchester)
	A549	NSCLC	Human	Xenograft^a^	CBA nu/nu mouse (University of Manchester)
	HCT116	Colorectal	Human	Xenograft^a^	CBA nu/nu mouse (University of Manchester)
	A2870	Ovarian carcinoma	Human	Xenograft^a^	CBA nu/nu mouse (University of Manchester)
	U87	Glioblastoma	Human	Xenograft^a^	CBA nu/nu mouse (University of Manchester)
	FTC113	Follicular thyroid carcinoma	Human	Xenograft^a^	CBA nu/nu mouse (University of Manchester)
	KHT	Sarcoma	Murine	Syngeneic autograft^a^	C3H mouse (University of Manchester)
Radboudumc Nijmegen	CC531	Colorectal	Rodent	Syngeneic liver metastasis^f^	Wag/Rij rats (Charles River)

^a^Subcutaneous tumours

^b^[15]

^c^Hingorani SR, Wang L, Multani AS, Combs C, Deramoudt TB, Hruban RH et al. Trp53R172H and KRASG12D cooperate to promote chromosomal instability and widely metastatic pancreatic ductal adenocarcinoma in mice. Cancer cell. 2005;7(5):469–483

^d^Spontaneous tumours arising in the pancreas

^e^Orthotopic tumours (mammary fat-pad implant)

^f^Tumours created by intra-hepatic injection

### Sample collection

Blood was collected by cardiac puncture into heparinized syringes and stored on ice until separation of the plasma by centrifugation at 4 °C. Plasma separation was performed within 30 min of blood collection in order to minimise thymidine degradation, which occurs in whole blood at 4 °C (Additional file 1: Figure S1). The plasma was frozen immediately and stored at −80 °C. Tumour and tissue samples were taken under terminal anaesthesia and frozen immediately in liquid nitrogen prior to storage at −80 °C. Only viable tumour tissue was sampled. Samples were couriered on dry ice to Cambridge for assay of thymidine content.

### Thymidine assay

Stable isotope labelled ^2^H_3_-thymidine (D3-TdR; Carbosynth Ltd, UK) was used as an internal standard in the liquid chromatography-mass spectrometry (LC-MS/MS) thymidine assay in place of the iodo-deoxyuridine standard used by Li et al. [8]. Other reagents were obtained from Sigma Aldrich (thymidine) and Fisher (Optima grade formic acid, acetonitrile and water).

All sample preparation was carried out on ice to minimise enzymatic degradation of thymidine. For plasma samples, the internal standard (D3-TdR, 100 ng/mL) was added to 50–100 μL aliquots of plasma, followed by 300 μL of ice-cold acetonitrile. After vortex mixing and centrifugation (3000*g*, 10 min), the supernatant was transferred to a 96-well plate and evaporated to dryness. For tumour and tissue samples, a minimum of 10 mg was required. Thymidine was extracted by homogenising tissue in ice-cold acetonitrile (50 % *v*/*v*) in a Precellys 24 tube homogeniser (50 mg/mL of tissue/homogenate). Aliquots of 50 or 100 μL were then subjected to the same extraction procedure as used for plasma. Samples were reconstituted in 100 μL water prior to analysis by LC-MS/MS.

For plasma analysis, human plasma was used as the control matrix for the calibration standards. Human plasma contains approximately 100 times less endogenous thymidine than mouse plasma and was further depleted of thymidine by incubation for 2 h at 37 °C, as described by Li et al. [8]. For tissue analysis, water was used as the control matrix. Calibration standards ranged from 1 to 500 ng/mL (plasma) and 0.08 to 40.8 μM (tissue).

The LC system used an Agilent 1290 pump with auto-injector, 4 °C sample cooler and 40 °C column heater. Analyte separation was performed on a Waters ACQUITY UPLC™ HSS T3 1.8 μm 50 × 2 mm i.d. column. The mobile phases were 0.1 % formic acid in water (mobile phase A) and 0.1 % formic acid in acetonitrile (mobile phase B) delivered at 0.6 mL/min. The gradient programme started with 100 % A for 0.5 min, decreasing to 60 % over 2 min, a further decrease to 10 % for 0.01 min, held at 10 % B for 0.5 min then increased to 100 % A for 0.01 min then held at 100 % for 3 min, to give a total run time of 6 min.

Mass spectrometry was performed with an ABSciex 6500 fitted with APCI probe operating in negative ion mode with a source temperature of 350 °C. Compound optimization was performed manually using Analyst software V1.6.2 (Sciex) following infusion of reference standards of thymidine and D3-TdR. Data was processed and integrated using Multiquant software V2.1.1 (Sciex). The most abundant transition was found to be fragmentation of the formate adduct to the molecular ion giving rise to the following selective reaction monitoring transitions of 287→241 and 290→244 for thymidine and D3-TdR, respectively.

### [^18^F]FLT PET imaging and data analysis

[^18^F]FLT PET scans were conducted at four centres (CI, AZ, IC and WMIC) on three different preclinical PET scanners (Inveon, Siemens Healthcare, UK; NanoScan PET/CT, Mediso, Hungary; and quadHIDAC, Oxford Positron Syst., UK). See Table 2 for details of PET scanners and methodology. All animals were anaesthetised using isoflurane in 100 % oxygen, and body temperature was maintained by heating. To minimise systematic differences in quantification, three measures were taken. Firstly, robust quality control measures were put in place at each centre. In particular, each centre adhered to their manufacturer’s recommended quality control schedule, including quantification standards at minimum six monthly intervals. Secondly, the tumour maximal intensity voxel was used because it is largely operator independent, so it controlled for subjective differences in region of interest drawing between centres. Thirdly, a ratiometric analysis, tumour-to-liver ratio (TTLmax) was used as the primary parameter. This minimised the influence of differences in hardware and reconstruction methods on absolute quantification and provided a relative measure of [^18^F]FLT uptake. In murine liver, [^18^F]FLT is not metabolised and can therefore be used as a reference organ [10]. Secondary parameters TTLmean, SUVmax and SUVmean were also compared to thymidine and plasma levels. Uptake parameters were determined at 60 ± 5 min after intravenous [^18^F]FLT injection. As the full range of parameters was not available for all 22 tumour models, data from only 14 models were used in the correlation of thymidine concentration with imaging data.

**Table 2:** Summary of imaging parameters at each centre

Research Centre	Injected dose (average, MBq)	Measurement time	PET scanner	Reconstruction method
CRUK Cambridge Institute (CI)	8.3	60–65 min	NanoScan PET/CT pre-clinical PET scanner (Mediso)	3D OSEM
AstraZeneca (AZ)	8.3	50–60 min	Inveon Siemens PET scanner	2D FBP
Imperial College London (IC)	3.7	50–60 min	Inveon Siemens PET scanner	2D OSEM
WMIC Manchester	9.5	57.5–62.5 and 55–65 min (FCT113 only)	quadHIDAC small animal PET scanner (Oxford Positron Systems) or Inveon Siemens PET scanner (U87 and FCT113 only)	OPL-EM or 3D-OSEM/MAP

### Tracer production

[^18^F]FLT was manufactured according to Ph.EU [11] specifications at the cyclotron centres available to the partners. The radiochemical purity in all the studies was more than 95 %.

### Statistics

Data were expressed as mean ± one standard deviation (SD), unless stated otherwise. The significance of comparison between two data sets was determined using the unpaired, two-tailed Student *t* test with Welch’s correction (Prism v6.0 software, GraphPadSoftware) and differences were considered significant if *p* ≤ 0.05. Mean [^18^F]FLT uptake value and mean thymidine concentration were determined in cohorts of mice, where each cohort was defined by tumour type and study centre. Correlations between cohorts were performed on mean data and assessed by the Spearman correlation coefficient (Prism v6.0 software, GraphPadSoftware).

## Results

### Performance of thymidine assay

**Fig. 1:**
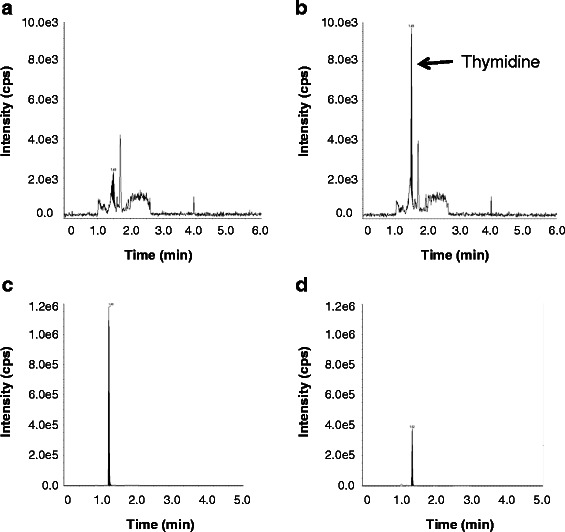
Examples of chromatograms. **a** Blank control plasma; thymidine-depleted human plasma was used as the control matrix. Human control plasma spiked with 2 ng/ml (8.3 nM) thymidine. **c** Typical mouse plasma sample. **d** Typical mouse tumour sample

Assay performance was assessed by using the EMA Guidelines on bioanalytical method validation (EMEA/CHMP/EWP/192217/2009) as a guide to determining the linearity, precision, accuracy, matrix effects and recovery. Linearity was tested using eight non-zero standards with the back-calculated concentration of each standard value not exceeding ±15 % of the theoretical value (±20 % at the lower limit of quantification (LLOQ)). The precision and accuracy were assessed by replicate analysis (*n* = 5) of quality control (QC) samples at four different concentrations. Recovery was assessed by comparing the peak response ratios of analytes spiked before and after extraction with those spiked at the same concentrations. Examples of chromatograms are shown in Fig. 1. Recovery of both thymidine and D3-TdR from plasma was 100 %. The results for precision and accuracy of the assay are summarised in Table 3.

**Table 3:** Precision and accuracy of plasma thymidine assay; acceptance criteria ±15 % for CV and RE (±20 % at LLOQ)

QC	Theoretical thymidine concentration (ng/mL)	Calculated thymidine mean concentration (ng/mL, *n* = 6)	CV (%)	RE (%)
LLOQ	1	0.99^a^	19	−0.9
Low	3	2.81	10	−6.4
Medium	40	47	13	−5.4
High	450	462	1.7	2.6

*LLOQ* Lower limit of quantification, *CV* coefficient of variation, *RE* relative error

^a^ *n* = 5

The method was shown to be robust and reproducible, with a lower detection limit of 1 ng/mL (4.1 nM). Any assay for which the accepted control criteria were not met was discarded and the assay repeated.

### Plasma and other normal tissue thymidine concentrations vary in different rodents

**Fig. 2:**
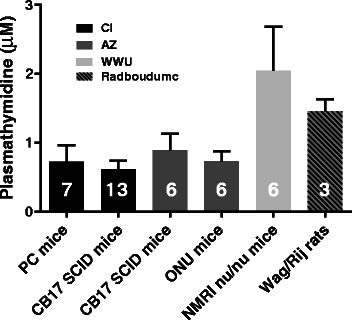
Plasma thymidine concentrations varied between different rodent species and mouse strains. Data are for non-tumour bearing animals from four centres; the centres housing the animals are indicated. See Table 1 for PC mouse genotype. *Error bars* show ± SD; numbers of animals are indicated on the *columns*

Plasma samples were collected from five mouse strains and one rat strain that are used routinely in preclinical studies. Plasma thymidine concentrations in non-tumour-bearing mice varied threefold between strains, from 0.61 ± 0.12 μM for CB17 SCID mice housed at the CI to 2.04 ± 0.64 μM for NMRI nude mice housed at WWU (Fig. 2). Interestingly, there was a significant difference between the plasma thymidine concentrations in the CB17 SCID strain housed at two different centres, namely 0.61 ± 0.12 μM for CB17 SCID mice at the CI and 0.89 ± 0.24 μM for CB17 SCID mice at AZ (*p* = 0.003). The variation in thymidine concentrations between strains at a single centre was not significant. The thymidine concentration in Wag/Rij rat plasma was within the range observed for mice.

**Fig. 3:**
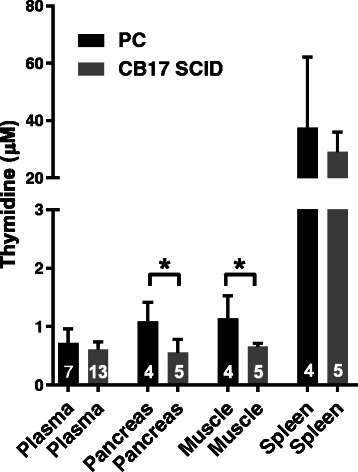
Plasma and normal tissue thymidine concentrations in PC (*black columns*) and CB17 SCID (*grey columns*) mice at CI. Pancreas was measured as the corresponding normal tissue for all CI pancreatic tumour models. See Table 1 for PC mouse genotype. Pancreas and muscle thymidine concentrations were significantly higher in PC than in CB17 SCID mice (*p ≤ 0.05). *Error bars* show ± SD; numbers of animals are indicated on the *columns*

Muscle, spleen and pancreas thymidine concentrations were measured in non-tumour-bearing mice in the PC and SCID mouse strains used at the CI (Fig. 3). Plasma, muscle and pancreas all showed thymidine concentrations between 0.55 ± 0.23 μM and 1.14 ± 0.40 μM, while concentrations in the spleen were approximately 40-fold higher. Thymidine concentrations were higher in PC than in SCID mice in both pancreas (*p* = 0.02) and muscle (*p* = 0.03) but were comparable in plasma.

### Different tumour models showed a wide range of thymidine concentrations

**Fig. 4:**
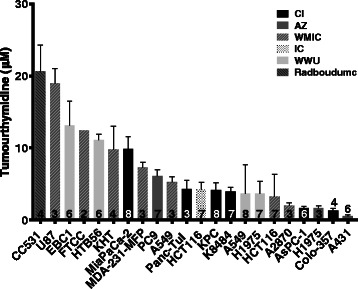
Tumour thymidine concentrations were very variable across tumour models, ranging from 0.54 ± 0.17 μM to 20.04 ± 3.65 μM. *Column fill* indicates the centre supplying the tumour samples. *Error bars* show ± SD; numbers of tumours are indicated on the *columns*. See Additional file 1: Figure S6 and ESM Table (a) for the plotted values and the median, maximum, minimum, first and third quartiles

Tumour thymidine concentrations were measured in a panel of 22 different tumours, including three xenografts grown from the same cell line but in two different centres (A549 at WMIC and WWU; HCT116 at IC and WMIC; H1975 at WWU and AZ). The tumour thymidine concentrations ranged from between 0.54 ± 0.17 μM for the A431 xenograft to 20.04 ± 3.65 μM for the syngeneic rat metastasis model CC531 (Fig. 4). The xenografts grown at two different centres showed comparable thymidine concentrations except for H1975 tumours, where thymidine in H1975 tumours at AZ was 1.64 ± 0.29 μM while for tumours grown at WWU, it was 3.63 ± 1.73 μM (*p* = 0.018). For A549, the WWU tumour thymidine concentration was 3.69 ± 3.96 μM, which was similar to the value of 5.25 ± 0.73 μM for the same tumours grown at WMIC. HCT116 tumours at IC and WMIC also had similar concentrations of 4.23 ± 0.97 and 3.27 ± 3.06 μM, respectively. There was no difference between the genetically engineered spontaneous KPC tumours and the syngeneic transplanted K8484 tumours.

### Tumours can affect plasma thymidine concentrations

**Fig. 5:**
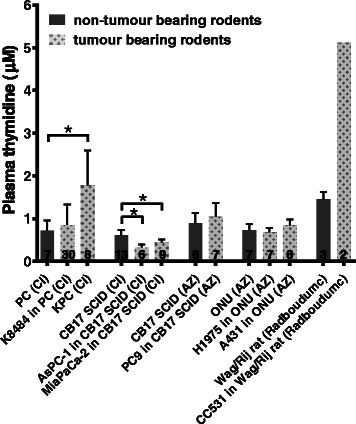
The presence of some tumours, but not all, affected plasma thymidine levels. Plasma thymidine levels are shown for five hosts, with or without tumours. PC mice were hosts for the K8484 tumour and were the non-tumour-bearing cage mates of mice that develop KPC tumours; PC mice lack the *KRAS* mutation present in the KPC mice and hence do not develop tumours. Columns are fill-coded to indicate the presence or absence of a tumour. *Error bars* show ± SD; numbers of animals are indicated on *columns*; the *asterisk* (*) indicates significance (*p* ≤ 0.05)

The presence of the LoVo subcutaneous xenograft has been associated with lower plasma thymidine concentrations in CD1 nu/nu mice [12]. However, for the three centres able to provide plasma samples from animals with and without tumours, this relationship was not consistent. Plasma thymidine was significantly lower in SCID mice with AsPC-1 (*p* < 0.0001) or MiaPaCa-2 tumours (*p* = 0.0016) than in non-tumour bearing mice, but higher in mice with KPC tumours than in the PC mice, which lack the KRAS mutation and do not develop spontaneous tumours (*p* = 0.006). The plasma thymidine concentration in tumour-bearing rats was also more than three times higher than that in naïve animals (Fig. 5).

### [^18^F]FLT uptake does not correlate with tumour thymidine concentrations

**Fig. 6:**
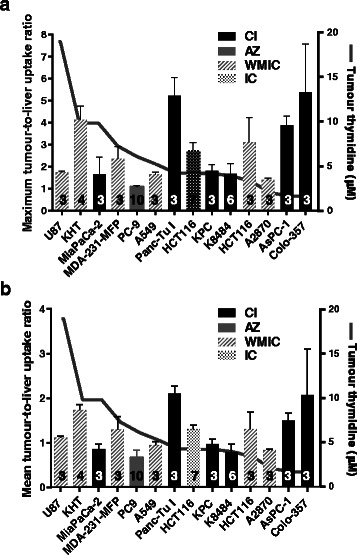
Tumour thymidine concentrations were not correlated with [^18^F]FLT uptake. Tumour-to-liver uptake (TTL) ratios ± SD are shown as a bar chart superimposed on a plot of the respective tumour thymidine concentrations. *Left axes* show uptake ratios; *right axes* show thymidine concentrations. **a** TTLmax. **b** TTLmean. Numbers of animals are indicated on the *columns*. Column fill indicates the centre supplying the tumour samples. See Additional file 1: Figure S7 and ESM tables (a), (b) and (c) for the plotted values and the median, maximum, minimum, first and third quartiles

For 14 of the 22 tumour models studied, the ratio of TTL had been determined at a comparable time of 60 ± 5 min after [^18^F]FLT injection. In this data set, TTLmax varied fivefold from 1.01 ± 0.19 to 5.22 ± 0.8 and TTLmean varied threefold from 0.67 ± 0.17 to 2.1 ± 0.2. There was no correlation between the PET measurements, TTLmax or TTLmean, and tumour thymidine concentrations (Fig. 6). This also held true when comparing SUVmax and SUVmean with tumour thymidine concentration (Additional file 1: Figure S2). Correlation plots for TTLmax, TTLmean, SUVmax and SUVmean with tumour thymidine are shown in Additional file 1: Figure S3; none of these correlations reached statistical significance. However, there was evidence that the data were consistent between sites. [^18^F]FLT uptake and tumour thymidine concentrations were measured in HCT116 tumours at IC and WMIC, where the former was measured using two different scanners (Inveon Siemens PET scanner and quadHIDAC small animal PET scanner, respectively). Both sites obtained very similar maximum and mean tumour-to-liver uptake ratios (maxTTL 2.74 ± 0.36 (IC) and 3.1 ± 1.13 (WMIC); meanTTL 1.31 ± 0.36 (IC) and 1.31 ± 0.38 (WMIC)) and similar thymidine concentrations (4.23 ± 0.97 μM (IC) and 3.27 ± 3.06 μM (WMIC)).

## Discussion

The strength of this study is that data were collected from multiple centres, allowing a more thorough investigation into the relationship between plasma and tumour thymidine concentrations and [^18^F]FLT uptake than has been possible previously. Plasma thymidine concentrations of non-tumour-bearing animals varied threefold between different strains (from 0.61 ± 0.12 μM for SCID mice at the CI to 2.04 ± 0.64 μM for NMRI nude mice at WWU), while variation in thymidine concentrations between strains at a single centre was low (Fig. 2). Differences between mouse strains might be expected; however, differences between the same strain at different centres, namely between SCID mice at CI and AZ, were more surprising. This might reflect differences in genetic background in the same strain from different sources, although local factors at the different centres may also be important. There was no significant difference in plasma concentrations between the two strains of mice housed at the CI (PC and SCID mice) or between two strains housed at AZ (SCID and nude mice). Most modern animal units have broadly similar environmental conditions; however, rodent diet from different suppliers may have an effect. In the present study, standardisation of diets across a large range of centres and countries was not possible due to logistical constraints. The CI diet is rich in protein and fat, whereas the AZ diet contains much lower levels of both, but is enriched in vitamins. The AZ diet contains approximately six times more folic acid than the CI diet, which could contribute to the difference in plasma thymidine concentrations [13]. Although, there is no published evidence that the source of nutrients influences plasma thymidine concentrations, gut microbiota are influenced by the genetic background of the host and diet; this can lead to differential breakdown of dietary components, which may provide a possible explanation for the differences in plasma thymidine concentrations [14] (Fig. 2).

While plasma concentrations in non-tumour-bearing animals were found to vary only 3-fold, there was a 40-fold range in tumour thymidine concentrations in the panel of 22 tumour models studied, from 0.54 ± 0.17 μM in the A431 xenograft to 20.04 ± 3.65 μM in the rat colon carcinoma liver metastasis model CC531 (Fig. 4). However, there was no obvious clustering of, for example, murine tumours derived from xenografts or xenograft tumours with the same tissue of origin. The CI pancreatic tumour xenografts MiaPaCa-2, Panc-Tu 1, AsPC-1 and Colo-357 showed a wide range of thymidine concentrations, ranging from 1 to 10 μM and of the four non-small cell lung cancer (NSCLC) xenografts studied at WWU, EBC1 and HTB56 had relatively high thymidine concentrations (12 μM), while A549 and H1975 had lower values (4 μM). In contrast, the spontaneous KPC pancreatic adenocarcinoma at the CI had similar thymidine concentrations to its transplanted allograft counterpart, K8484. This was despite differences in tumour architecture and stroma [15], which perhaps suggests that tumour thymidine concentrations within the same strain of mice are determined primarily by tumour genetics rather than site of growth within the host. The plasma thymidine concentration in KPC tumour-bearing mice was 2.8-fold higher than that in PC mice while the plasma thymidine concentration in K8484 tumour-bearing mice was not significantly different. In addition, the presence of CC531 tumours resulted in a 3.8-fold increase in plasma thymidine concentration. In contrast, AsPC-1 and MiaPaCa-2 xenografts caused a small but significant decrease in plasma thymidine concentration while the other three xenografts had no effect. In summary, in five out of the eight tumour models studied, the tumour affected the plasma thymidine level of their respective hosts. Plasma and tumour thymidine concentration appeared not to be correlated in those animals in which both concentrations were measured (Additional file 1: Figure S8).

The most striking result in this study was the lack of correlation between tumour thymidine concentrations and [^18^F]FLT uptake across 14 different tumour models and 4 centres (Fig. 6 and Additional file 1: Figure S2 and Figure S3). One cannot exclude that differences between the three PET scanners and reconstruction algorithms used have introduced variability into the dataset. However, by employing regular quality control measures and by using tumour-to-tissue ratios rather than SUV as the primary parameter describing uptake variability due to technical differences is expected to be minimised. Previous work has shown a negative correlation between [^18^F]FLT uptake and tumour thymidine concentrations in smaller cohorts from single centres [5, 6]. The current work however implies that if there is competition between thymidine in the tumour and [^18^F]FLT, tumour thymidine concentration alone is not sufficient to predict [^18^F]FLT uptake. In a subset where tumour thymidine and [^18^F]FLT uptake were available for individual mice from a single centre, there was no negative correlation (Additional file 1: Figure S9). This was also observed in the models studied by McKinley et al. [7], highlighting the complexity of factors affecting [^18^F]FLT uptake. Variable effects of metabolism, clearance, cell membrane transporters, thymidine kinase 1 (TK1) activity, dephosphorylation and inflammatory infiltration on [^18^F]FLT uptake all probably play a part to different degrees [16].

Zhang et al. [5] showed that in the HCT116 xenograft, [^18^F]FLT uptake was inversely correlated with plasma thymidine concentration, which was delivered by a mini-pump. However, in the six tumour models where plasma samples were available, we found no correlation between endogenous plasma thymidine concentrations and [^18^F]FLT uptake (Additional file 1: Figure S4). To determine if a tumour-to-plasma thymidine concentration gradient had an effect on [^18^F]FLT uptake in these models, we also considered the differential between the plasma and tumour thymidine concentrations, but similarly found no correlation between this parameter and tumour-to-liver uptake ratio (Additional file 1: Figure S5).

Despite plasma thymidine concentrations being higher in rodents as compared to humans [8], the lack of a simple relationship between plasma or tumour thymidine concentration and [^18^F]FLT uptake in the mice studied here suggests that this may also be the case in patients. Therefore, future clinical studies examining the performance of [^18^F]FLT, particularly where [^18^F]FLT has failed to report on tumour proliferation, should look beyond plasma thymidine concentration as a possible explanation for differences in tumour uptake.

## Conclusions

Tumour thymidine concentrations were not correlated with [^18^F]FLT uptake in a broad range of preclinical tumour models. There was no evidence from this study that a simple thymidine test, either on plasma or tumour samples, could be used to predict the value of an [^18^F]FLT PET scan for individual patients.

This study also underlines the need for a more detailed investigation of the determinants of thymidine metabolism, such as the expression and activity of the enzymes TK1, thymidine phosphorylase and thymidylate synthase, in a similarly wide range of tumour models.

## Ethical approval

All applicable international, national and/or institutional guidelines for the care and use of animals were followed. All procedures performed were in accordance with the ethical standards of the institution or practice at which the studies were conducted. Further information is given in the “Methods” section.

## Abbreviations

[^18^F]FLT, 3′-deoxy-3′-[^18^F] fluorothymidine; AZ, AstraZeneca; CI, CRUK Cambridge Institute; D3-TdR, ^2^H_3_-thymidine; IC, Imperial College London; LC-MS/MS, liquid chromatography-mass spectrometry; LLOQ, lower limit of quantification; NSCLC, non-small cell lung cancer; PET, positron emission tomography; QC, quality control; Radboudumc, Radboud University Medical Centre; TK1, thymidine kinase 1; TTL, tumour-to-liver uptake ratio; WMIC, Wolfson Molecular Imaging Centre Manchester; WWU, Westfälische Wilhelms-Universität

## Additional file

Additional file 1: Supplementary material. (PDF 647 kb)

## Footnotes

1 The QuIC-ConCePT (Quantitative Imaging in Cancer: Connecting Cellular Processes with Therapy) project is an IMI funded project which aims to provide drug developers with tools, imaging biomarkers, that can show earlier and more accurately how patients’ tumours respond to drugs in cancer clinical trials (http://www.quic-concept.eu/).
