# Correction to: The relationship between endogenous thymidine concentrations and [^18^F]FLT uptake in a range of preclinical tumour models

**DOI:** 10.1186/s13550-017-0349-1

**Published:** 2017-12-15

**Authors:** Kathrin Heinzmann, Davina Jean Honess, David Yestin Lewis, Donna-Michelle Smith, Christopher Cawthorne, Heather Keen, Sandra Heskamp, Sonja Schelhaas, Timothy Howard Witney, Dmitry Soloviev, Kaye Janine Williams, Andreas Hans Jacobs, Eric Ofori Aboagye, John Richard Griffiths, Kevin Michael Brindle

**Affiliations:** 1 Cancer Research UK Cambridge Institute, University of Cambridge, Cambridge, UK; 2 Wolfson Molecular Imaging Centre, Manchester Pharmacy School, University of Manchester, Manchester, UK; 3 Personalised Healthcare and Biomarkers, AstraZeneca, Alderley Park, Macclesfield UK; 4 Department of Radiology and Nuclear Medicine, Radboud University Medical Centre, Nijmegen, The Netherlands; 5 European Institute for Molecular Imaging (EIMI), Westfälische Wilhelms-Universität (WWU), University Hospital of Münster, Münster, Germany; 6 Comprehensive Cancer Imaging Centre, Imperial College London, London, UK; 7 CRUK-EPSRC Cancer Imaging Centre in Cambridge and Manchester, Cambridge, UK; 8 Comprehensive Cancer Imaging Centre, Imperial College London, London, UK; 9 Positron Emission Tomography Research Centre, University of Hull, Hull, UK; 10 UCL Centre for Advanced Biomedical Imaging, University College London, London, UK; 11 Cancer Research UK Cambridge Institute, Li Ka Shing Centre, Cambridge, CB2 0RE UK

## Correction

Unfortunately, the original version of Figs. 4, 5 and 6b in the article [1] contained errors in the *n* numbers as indicated on the columns. Please note that column heights and error bars in the original figures and data in the ESM tables are correct and statistical tests are valid. These corrections do not affect any results or conclusions in this article.

The correct *n* numbers are as follows:

Figure 4 (http://media.springernature.com/full/springer-static/image/art%3A10.1186%2Fs13550-016-0218-3/MediaObjects/13550_2016_218_Fig4_HTML.gif): ‘HCT116’ (IC) *n* = 8; ‘A549’ (WWU) *n* = 7; ‘HCT116’ (WMIC) *n* = 3; ‘H1975’ (AZ) *n* = 6.

Figure 5 (http://media.springernature.com/full/springer-static/image/art%3A10.1186%2Fs13550-016-0218-3/Media Objects/13550_2016_218_Fig5_HTML.gif): ‘ONU (AZ)’ *n* = 6; ‘H1975 in ONU (AZ)’ *n* = 6; ‘A431 in ONU (AZ)’ *n* = 6.

Figure 6b (http://media.springernature.com/full/springer-static/image/art%3A10.1186%2Fs13550-016-0218-3/MediaObjects/13550_2016_218_Fig6_HTML.gif): ‘PC9’ *n* = 13.
