# Supplementary material for: The relationship between endogenous thymidine concentrations and [18F]FLT uptake in a range of preclinical tumour models
Source: EJNMMI Res. 2016 Aug 11;6:63. doi: 10.1186/s13550-016-0218-3 (PMC4980847; doi:10.1186/s13550-016-0218-3)
Supplement: Supplementary file 1 — Supplementary material. (PDF 647 kb) [file 13550_2016_218_MOESM1_ESM.pdf]

Journal: EJNMMI Research

Title: The relationship between endogenous thymidine concentrations and [^{18}F]FLT uptake in a range of preclinical tumour models

Authors: Kathrin Heinzmann[1,8], Davina J Honess[1], David Y Lewis[1,7], Donna-Michelle Smith[1], Christopher J Cawthorne[2,9], Heather Keen[3], Sandra Heskamp[4], Sonja Schelhaas[5], Timothy H Witney[6,10], Dmitry Soloviev[1,7], Kaye J Williams[2,7], Andreas H Jacobs[5], Eric O Aboagye[6], John R Griffiths[1] and Kevin M Brindle[1,7]

Corresponding author: Kevin M Brindle, D. Phil., Cancer Research UK Cambridge Institute, Li Ka Shing Centre, Robinson Way, Cambridge, CB2 0RE, UK. E-mail: kmb1001@cam.ac.uk

ELECTRONIC SUPPLEMENTARY DETAILS

The authors are members of the QuIC-ConCePT Consortium whose participants included: AstraZeneca, European Organisation for Research and Treatment of Cancer (EORTC), University of Cambridge, University of Manchester, Westfälische Wilhelms-Universität Münster, Radboud University Nijmegen Medical Center, Institut National de la Santé et de la Recherche Médical, Stichting Maastricht Radiation Oncology “Maastro Clinic”, VUmc Amsterdam, King’s College London, Universitair Ziekenhuis Antwerpen, Institute of Cancer Research – Royal Cancer Hospital, Erasmus Universitair Medisch Centrum Rotterdam, Imperial College of Science Technology and Medicine, Keosys S.A.S., Eidgenössische Technische Hochschule Zürich, Amgen NV, Eli Lilly and Company Ltd, GlaxoSmithKline Research & Development Limited, Merck KGa, Pfizer Limited, F.Hoffmann - La Roche Ltd, Sanofi-Aventis Research and Development

ELECTRONIC SUPPLEMENTARY FIGURES

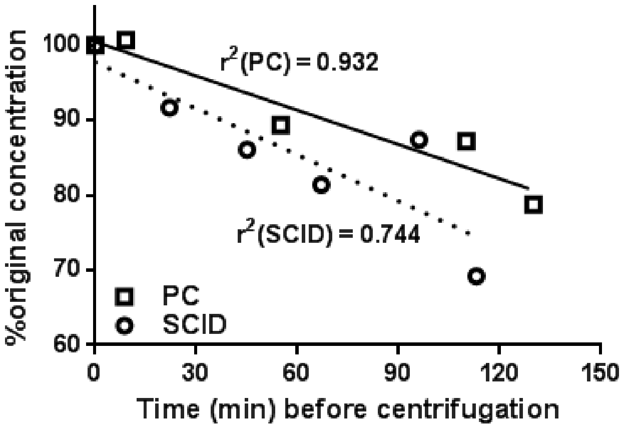

Fig. S1 Thymidine is not stable in murine blood kept on ice. Blood samples taken by cardiac puncture were split immediately into two cooled Eppendorf tubes. One was centrifuged and the plasma frozen immediately after separation. The other was kept on ice for a period of up to 130 minutes before centrifugation and plasma collection and freezing. The thymidine concentration of each sample kept on ice is expressed relative to the value in the portion of the sample that was processed immediately. Data are presented for plasma from both SCID (circles) and PC (squares) mice at the CI. See Table 1 for PC mouse genotype

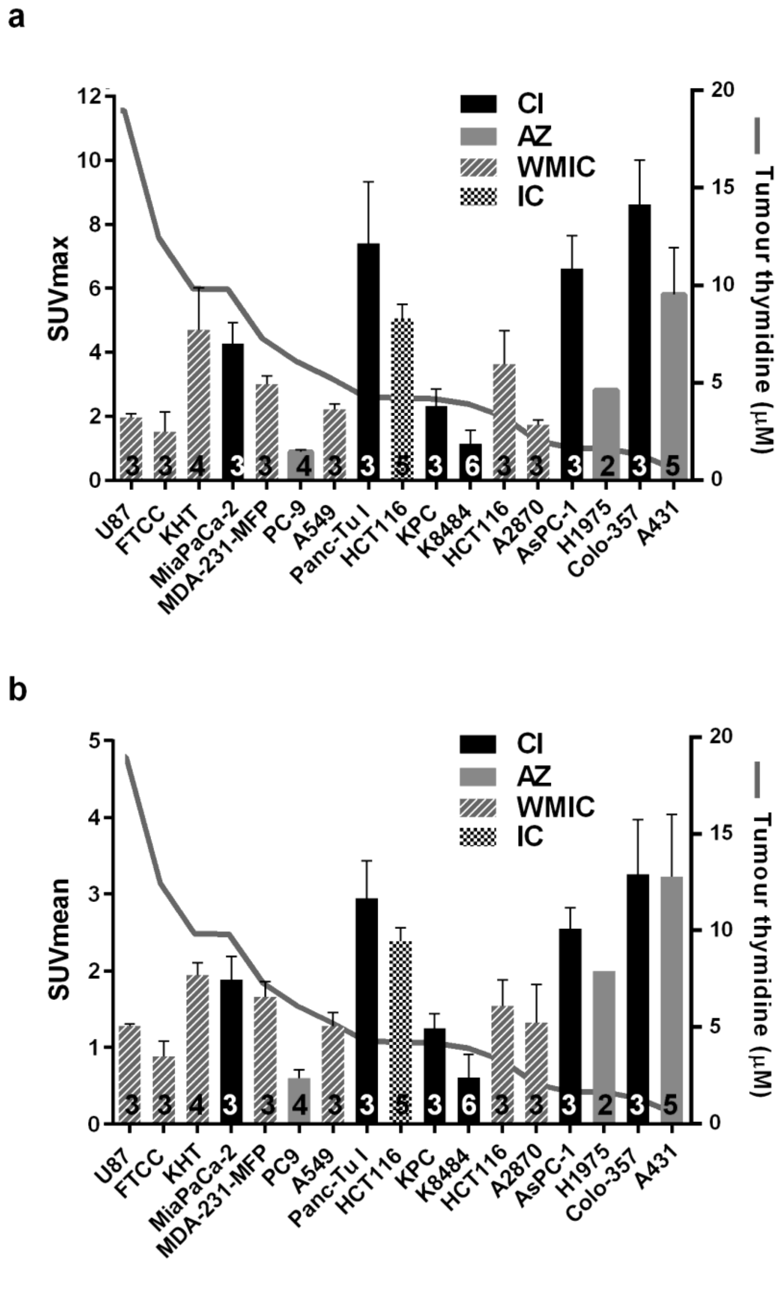

Fig. S2 There was no correlation between tumour thymidine concentration with either SUVmax (a) or (b) SUVmean. SUV data ± SD is shown as a bar chart superimposed on a plot of the respective thymidine concentrations. Left axes show uptake ratios; right axes show thymidine concentrations. Numbers of animals are indicated on the columns. Column fill indicates the centre supplying the tumour samples. Additional data (SUV and thymidine data for FTCC, H1975 and A431) were added here as the tumour-to-liver (TTL) uptake ratios for these tumours were not available

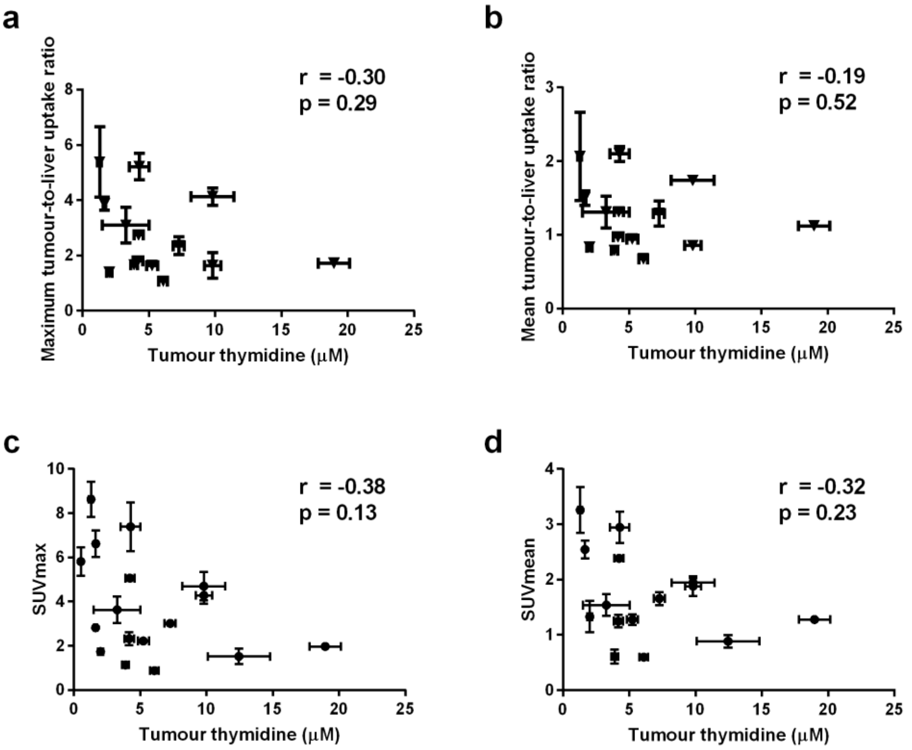

Fig. S3 Tumour thymidine concentrations were not correlated with either maximum (a and c) or mean [^{18}F]FLT uptake (b and d). Panels (a and b) show TTL ratios, panels (c and d) show SUV measurements. Data is expressed as mean ± SEM

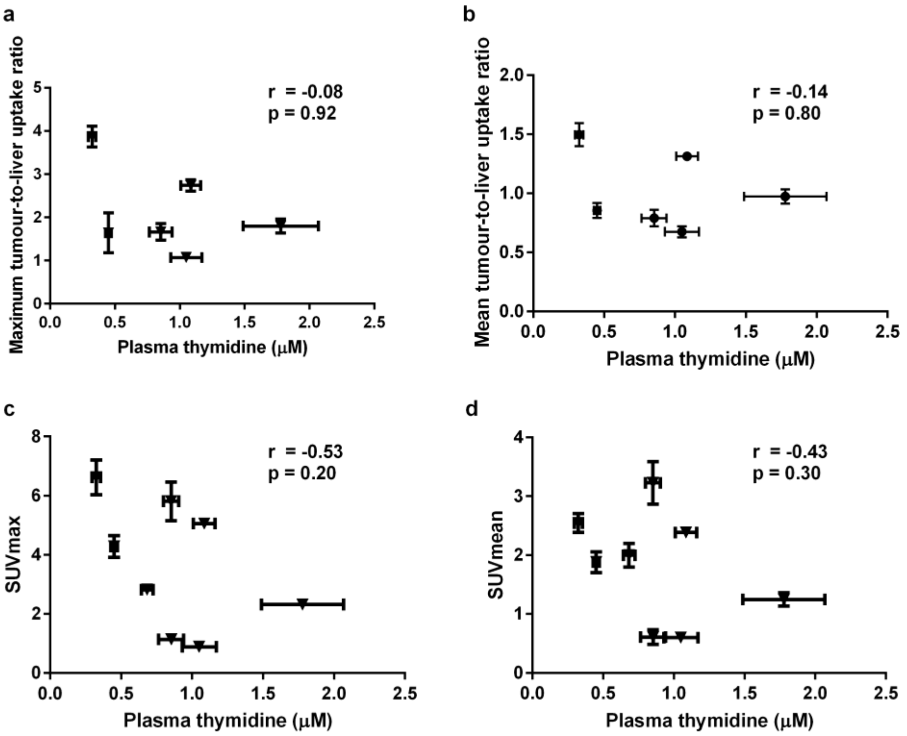

Fig. S4 There was no correlation between plasma thymidine concentration and either maximum (a) or mean (b) tumour-to-liver uptake ratio, or to SUVmax (c) or SUVmean (d). Data are expressed as mean ± SEM

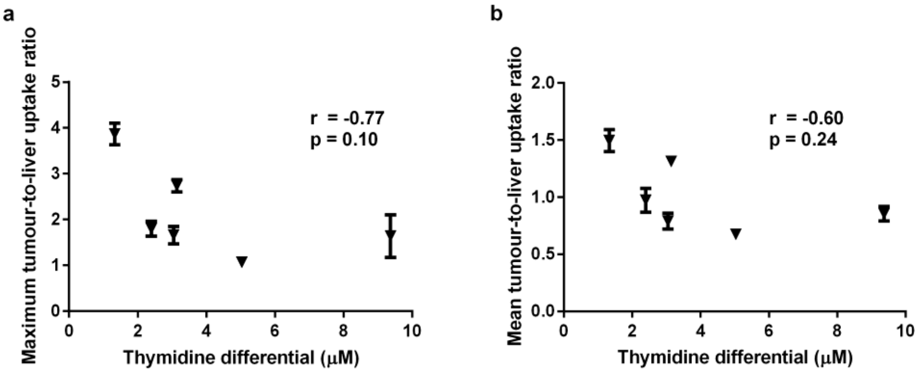

Fig. S5 There was no correlation between the difference in tumour and plasma thymidine concentrations and either the maximum (a) or mean (b) tumour-to-liver uptake ratio. The differential was calculated by subtracting the mean plasma thymidine concentration from the mean tumour thymidine concentration for each tumour model

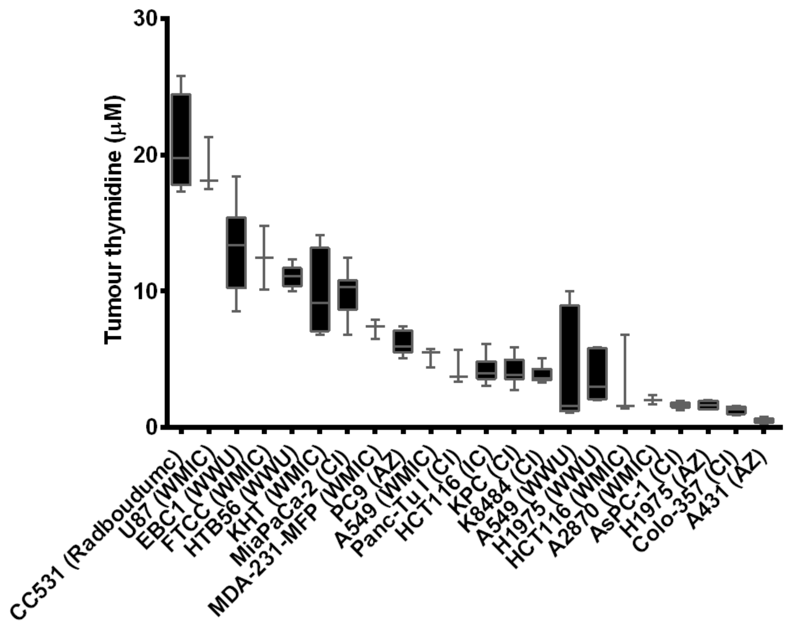

Fig. S6 Tumour thymidine concentrations varied across tumour models. The box and whisker plot shows the minimum, first quartile, median, third quartile and maximum

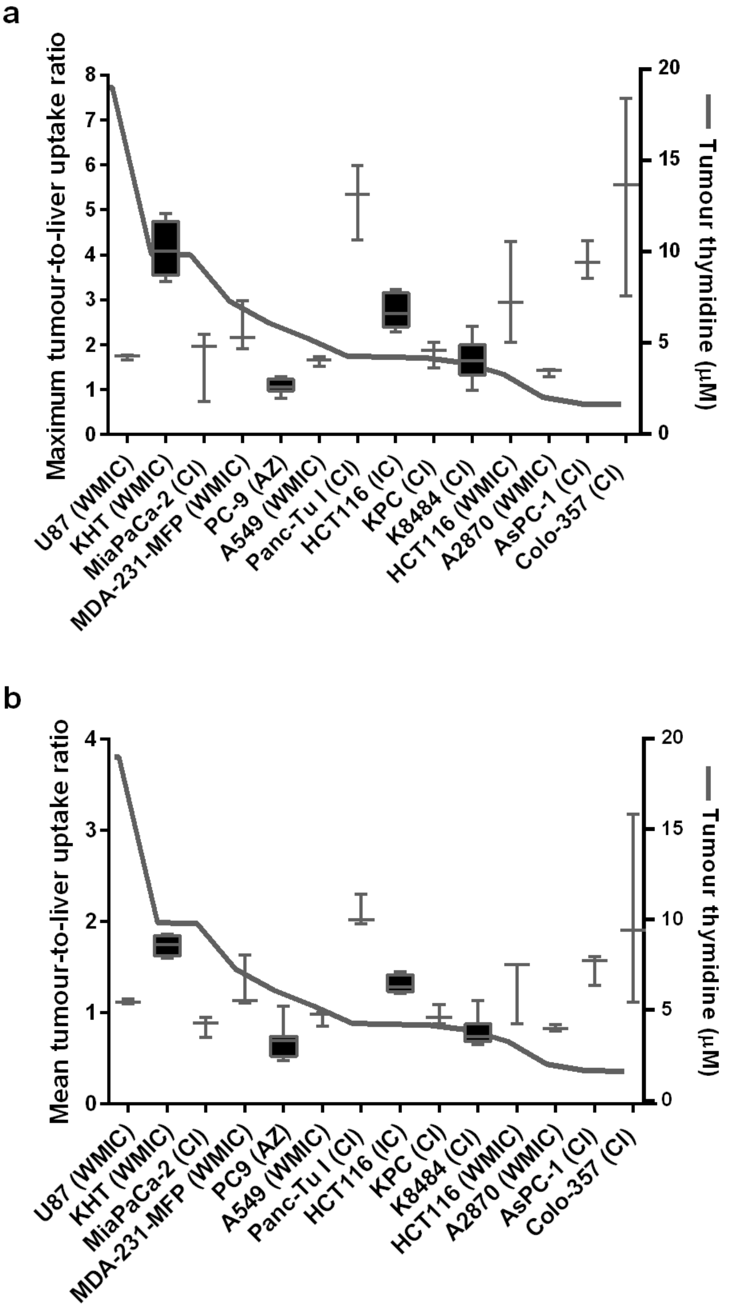

Fig. S7 Tumour thymidine concentrations were not correlated with [18F]FLT uptake. Tumour-to-liver (TTL) ratios are shown in a box and whisker plot displaying the minimum, first quartile, median, third quartile and maximum. The plot is superimposed on a plot of the respective tumour thymidine concentrations. Left axes show uptake ratios; right axes show thymidine concentrations. Panel (a) shows TTLmax, panel (b) shows TTLmean

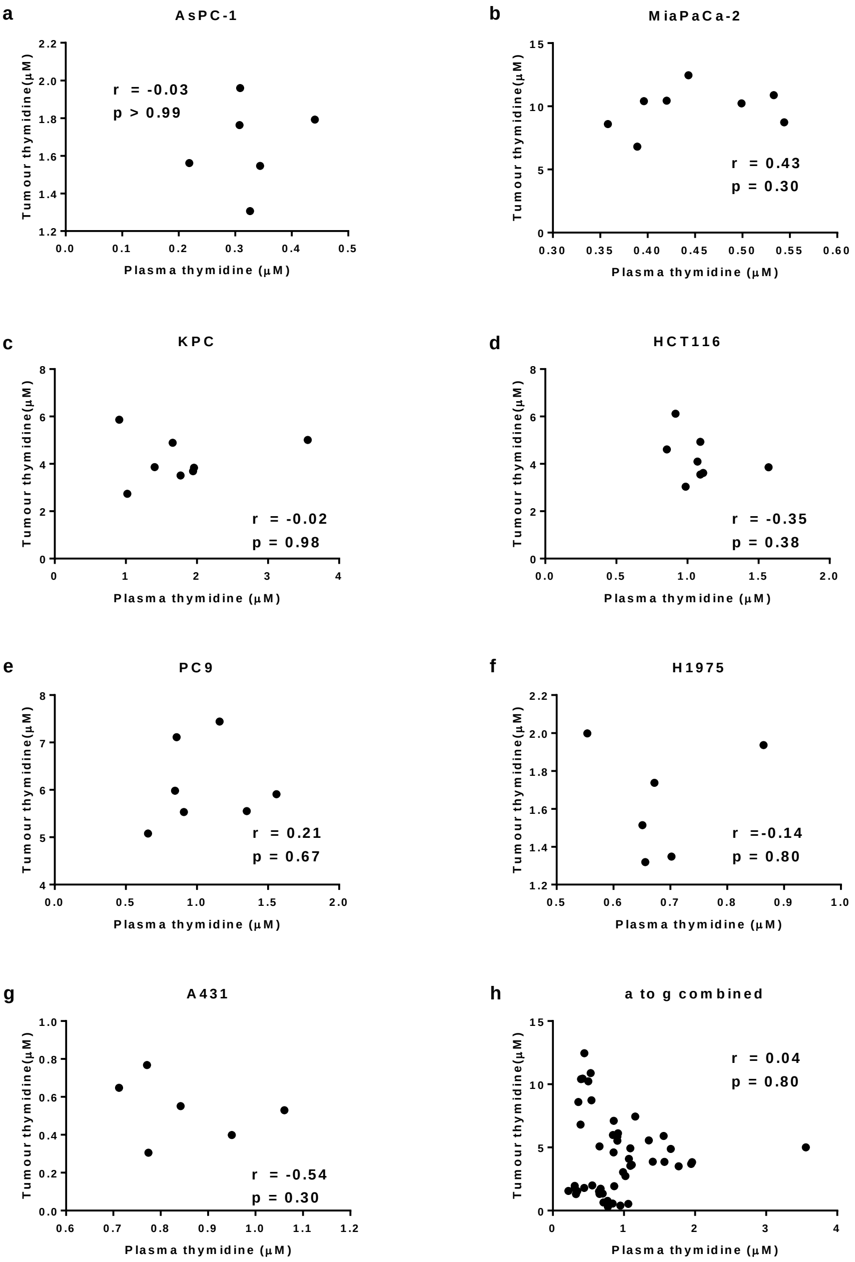

Fig. S8 Tumour thymidine concentrations were not correlated with plasma thymidine concentrations. Plasma and tumour thymidine concentrations were determined in the same animal. Data from the various tumour models are plotted individually (a to g) and with all data combined (h)

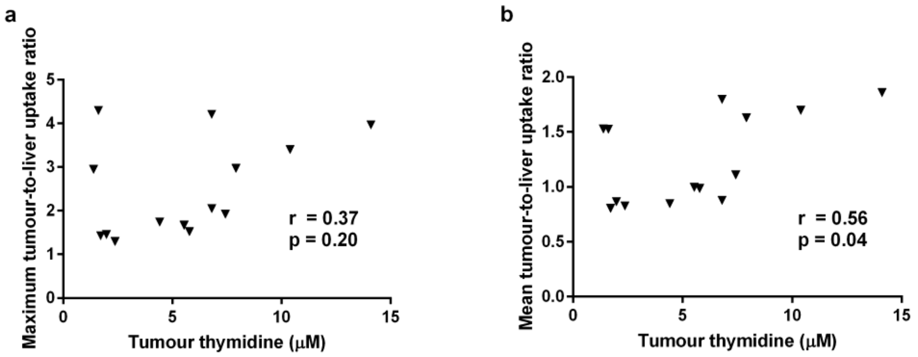

Fig. S9 Correlation plots of tumour thymidine concentrations and either maximum (a) or mean (b) tumour-to-liver uptake ratios for a subset of tumours where tumour thymidine and [^{18}F]FLT uptake were measured in the same animal (measurements made at WMIC).

ESM Tables (a,b and c) Values shown in Fig. 4, 6, S6 and S7 (Note: Percentiles are only quoted where n > 4)

(a) Tumour thymidine concentrations (μM)

	K8484 (CI)	KPC (CI)	AsPC-1 (CI)	MiaPaCa-2 (CI)	Colo-357 (CI)	Panc-Tu I (CI)	PC9 (AZ)	H1975 (AZ)	A431 (AZ)	HCT116 (IC)	A549 (WWU)
n	7	8	6	8	4	3	7	6	6	8	7
Mean	3.902	4.174	1.655	9.818	1.294	4.278	6.086	1.642	0.534	4.229	3.689
Std. Deviation	0.635	1.002	0.231	1.723	0.293	1.263	0.869	0.293	0.167	0.972	3.960
Minimum	3.310	2.735	1.306	6.802	0.885	3.390	5.080	1.319	0.305	3.040	1.100
25% Percentile	3.470	3.553	1.486	8.625			5.530	1.341	0.376	3.568	1.200
Median	3.619	3.849	1.662	10.32	1.368	3.720	5.910	1.626	0.541	3.980	1.600
75% Percentile	4.300	4.979	1.834	10.78			7.110	1.952	0.678	4.850	8.940
Maximum	5.107	5.863	1.960	12.45	1.556	5.723	7.440	1.998	0.768	6.120	9.980

(a) Tumour thymidine concentrations (μM)

	EBC1 (WWU)	HTB56 (WWU)	H1975 (WWU)	CC531 (Rad-boudumc)	MDA-231-MFP (WMIC)	A549 (WMIC)	HCT116 (WMIC)	A2870 (WMIC)	U87 (WMIC)	FTCC (WMIC)	KHT (WMIC)
n	6	6	7	4	3	3	3	3	3	2	4
Mean	13.13	11.08	3.629	20.65	7.277	5.247	3.267	2.020	18.97	12.45	9.808
Std. Deviation	3.361	0.796	1.727	3.650	0.7223	0.7259	3.062	0.3318	2.043		3.232
Minimum	8.500	10.00	2.000	17.30	6.490	4.420	1.390	1.710	17.50	10.10	6.800
25% Percentile	10.23	10.38	2.100								
Median	13.35	11.10	3.000	19.75	7.430	5.540	1.610	1.980	18.10		9.165
75% Percentile	15.40	11.70	5.800								
Maximum	18.40	12.30	5.900	25.80	7.910	5.780	6.800	2.370	21.30	14.80	14.10

(b) Maximum tumour-to-liver uptake ratios

	K8484 (CI)	KPC (CI)	AsPC-1 (CI)	MiaPaCa-2 (CI)	Colo-357 (CI)	Panc-Tu I (CI)	PC-9 (AZ)	HCT116 (IC)	MDA-231-MFP (WMIC)	A549 (WMIC)	HCT116 (WMIC)	A2870 (WMIC)	U87 (WMIC)	KHT (WMIC)
n	6	3	3	3	3	3	10	7	3	3	3	3	3	4
Mean	1.66	1.80	3.87	1.64	5.38	5.22	1.07	2.74	2.35	1.64	3.10	1.39	1.72	4.13
Std. Deviation	0.47	0.29	0.42	0.80	2.20	0.83	0.16	0.36	0.55	0.11	1.13	0.09	0.06	0.63
Minimum	0.992	1.49	3.48	0.73	3.09	4.34	0.796	2.29	1.92	1.52	2.05	1.29	1.65	3.40
25% Percentile	1.32						0.975	2.38						
Median	1.65	1.87	3.82	1.97	5.57	5.34	1.06	2.70	2.16	1.67	2.95	1.43	1.75	4.09
75% Percentile	2.00						1.23	3.15						
Maximum	2.40	2.05	4.31	2.23	7.48	5.99	1.28	3.23	2.97	1.74	4.29	1.46	1.77	4.93

(c) Mean tumour-to-liver uptake ratios

	K8484 (CI)	KPC (CI)	AsPC-1 (CI)	MiaPaCa-2 (CI)	Colo-357 (CI)	Panc-Tu I (CI)	PC9 (AZ)	HCT116 (IC)	MDA-231-MFP (WMIC)	A549 (WMIC)	HCT116 (WMIC)	A2870 (WMIC)	U87 (WMIC	KHT (WMIC)
n	6	3	3	3	3	3	13	7	3	3	3	3	3	4
Mean	0.790	0.972	1.496	0.854	2.067	2.099	0.716	1.312	1.291	0.944	1.310	0.831	1.120	1.739
Std. Deviation	0.174	0.106	0.1677	0.111	1.039	0.176	0.165	0.090	0.293	0.083	0.375	0.030	0.029	0.113
Minimum	0.650	0.878	1.304	0.732	1.119	1.978	0.475	1.215	1.109	0.848	0.878	0.804	1.094	1.602
25% Percentile	0.682						0.521	1.232						
Median	0.736	0.953	1.569	0.885	1.905	2.018	0.694	1.282	1.135	0.986	1.526	0.827	1.116	1.747
75% Percentile	0.877						0.742	1.417						
Maximum	1.132	1.086	1.614	0.946	3.178	2.300	1.071	1.452	1.629	0.997	1.527	0.864	1.151	1.859
